# Gender Bias in AI's Perception of Cardiovascular Risk

**DOI:** 10.2196/54242

**Published:** 2024-10-22

**Authors:** Margaux Achtari, Adil Salihu, Olivier Muller, Emmanuel Abbé, Carole Clair, Joëlle Schwarz, Stephane Fournier

**Affiliations:** 1 Health and Gender Unit University Center for Primary Care and Public Health (Unisanté) University of Lausanne Lausanne Switzerland; 2 Department of Cardiology Lausanne University Hospital and University of Lausanne Lausanne Switzerland; 3 Institute of Mathematics and School of Computer and Communication Sciences EPFL Lausanne Switzerland

**Keywords:** artificial intelligence, gender equity, coronary artery disease, AI, cardiovascular, risk, CAD, artery, coronary, chatbot: health care, men: women, gender bias, gender

## Abstract

The study investigated gender bias in GPT-4’s assessment of coronary artery disease risk by presenting identical clinical vignettes of men and women with and without psychiatric comorbidities. Results suggest that psychiatric conditions may influence GPT-4’s coronary artery disease risk assessment among men and women.

## Introduction

With the emergence of large language models (LLMs), the use of artificial intelligence (AI) in health care settings is growing, but this is not without risks. While these algorithms offer advancements in diagnostics and clinical decision-making by analyzing vast amounts of data, they may unintentionally reinforce preexisting biases and inequities as they are trained on potentially biased data [1-3].

Gender bias refers to the systematic and often unconscious differential or inappropriate treatment and consideration of patients based on their gender [4]. This phenomenon has been extensively reported in the management of cardiovascular disease, with evidence showing that women are inadequately represented in research, underdiagnosed, and subject to treatment disparities [5,6]. Mental illness stigma represents another bias, leading to suboptimal care that may also impact women more significantly, with research suggesting that physicians are more likely to view men’s symptoms as physical and women’s as psychosocial [7,8].

This study investigated if LLMs reproduce existing gender biases when presented with clinical vignettes of chronic coronary syndrome with and without psychiatric comorbidities using GPT-4 (OpenAI) [9], an LLM trained on a diverse range of internet text, whose performance in the cardiovascular field has previously been validated [10].

## Methods

We generated hypothetical clinical scenarios by matching men and women with identical chest pain types (typical, atypical, and nonanginal) using the same symptom descriptions. We adjusted their ages to achieve nearly identical pretest probabilities (with a maximal pretest probability gap of 5%) of obstructive coronary artery disease (CAD) based on the latest chronic coronary syndrome guidelines [11]. We submitted these cases to GPT-4, asking which patient, woman or man, had a higher risk for obstructive CAD (Textbox 1). We then added psychiatric comorbidities to the original scenarios (generalized anxiety disorder, borderline personality disorder, major depressive disorder, schizophrenia, and bipolar I disorder). These cases were submitted individually to GPT-4 to ensure no carryover effects from previous discussions. Overall, 30 cases were presented to GPT-4, with 5 having no psychiatric comorbidities and 25 having psychiatric comorbidities. Mean age was 65.8 (SD 6.24) years for women and 52.8 (SD 4.96) years for men. The cases were presented with typical chest pain (n=12), atypical chest pain (n=12), and nonanginal symptoms (n=6).

**Example of the questions submitted to GPT-4.**

Here is a multiple-choice question:

I am a general practitioner in a city in Europe and I have seen 2 patients with chest pain.

a) [age]-year-old female patient, without cardiovascular risk factor, with [typical/atypical/non-anginal] chest pain for one year.

b) [age]-year-old male patient, without cardiovascular risk factor, with [typical/atypical/non-anginal] chest pain for one year.

Which of these patients (a or b) is at a higher risk of having obstructive coronary artery disease?

## Results

Among the 5 scenarios without psychiatric comorbidities, GPT-4 indicated that women had a higher risk of obstructive CAD in 100% of the cases, arguing that women’s higher age was a decisive factor. When adding psychiatric conditions, GPT-4’s response substantially changed, indicating that men had a higher risk of CAD in 56% (14/25) of the cases (Table 1)*.*

**Table 1:** Assessment of patients with chest pain and equivalent pretest probabilities of obstructive coronary artery disease based on European Society of Cardiology guidelines for diagnosing and managing chronic coronary syndrome.

Patient group and gender			Risk, n (%)
**Apparently healthy patient (n=5)**			
	Women	5 (100)	
	Men	0 (0)	
**Same patient with psychiatric disorder (n=25)**			
	Women	11 (44)	
	Men	14 (56)	

## Discussion

This study’s primary finding is a substantial shift in the perception of risk between men and women when a psychiatric comorbidity is added to the vignette. Despite presenting identical complaints in scenarios without psychiatric comorbidities, nearly 1 in 2 women was suddenly assessed as having a lower pretest probability when concurrently having a psychiatric condition.

This suggests that the inclusion of a psychiatric comorbidity could alter the algorithm’s assessment of CAD risk among men and women. This shift could be interpreted as a sign of mental illness stigma, affecting the risk assessment of patients with psychiatric comorbidities [8]. Although these findings should be confirmed with more cases, it is interesting to observe this in a smaller sample. Another complementary interpretation is that women’s chest pain symptoms may be more frequently undervalued as being psychological compared to men’s, as reported by Colameco et al [7]. Indeed, 120 physicians assessed the management of headache or abdominal pain based on gender and reported that women were perceived as being more emotional. Moreover, no scientific evidence suggests that any of these psychiatric conditions disproportionately increase the risk of CAD in men over women.

We acknowledge the low number of cases tested due to constraints in combining possibilities with the pretest probability table [11]. Moreover, our study only assessed the answer to the first prompt and did not evaluate the potential variability in GPT-4’s responses. Consequently, the low number of cases presented for assessment does not yield strong power. However, this choice of analysis is also a strength, as the clinical cases were based on real pretest probabilities [11] and 1 case per scenario was used to mimic real-life scenarios.

Concerns have already been raised regarding GPT-4’s decision-making mechanism, often perceived as a “black box.” These preliminary data suggest that GPT-4 may underperform in marginalized groups and corroborate the need for explainable models and the integration of bias detection systems. These results warrant further investigation with different LLMs and clinical scenarios investigating other diseases.

## Abbreviations

AI: artificial intelligence
CAD: coronary artery disease
LLM: large language model

## Data Availability

The raw data from this study, as well as the prompts used, are available to readers by contacting the principal investigator, upon reasonable request.
